# Molecular Structure Regulation of Polyacrylamide-Based Drag Reducers on Solubility and Transient Gel-Layer Behavior: Molecular Dynamics and Experimental Study

**DOI:** 10.3390/gels12050369

**Published:** 2026-04-28

**Authors:** Ke Xu, Congcong Yu, Dingwei Weng, Yuetong Zhao, Jianchao Liu, Zhengxiong Su, Guanxin Zeng, Jing Long, Cunchuan Zheng

**Affiliations:** 1 College of Chemistry and Chemical Engineering, Southwest Petroleum University, Chengdu 610500, China; 18091574267@163.com (C.Y.); hw27315k@163.com (Y.Z.); lj73055@163.com (J.L.); 2 China Petroleum Exploration and Development Research Institute, Beijing 100083, China; wd63641@163.com; 3 China National Petroleum Corporation Tarim Oilfield Company Oil and Gas Technology Research Institute, Kuerle 841000, China; liujianc-tlm@petrochina.com.cn (J.L.); suzhx-tlm@petrochina.com.cn (Z.S.); 13881784961@163.com (G.Z.)

**Keywords:** polyacrylamide, molecular dynamics, dissolution rate, slippery water

## Abstract

This study aimed to clarify how molecular structure regulates the dissolution and transient gel-layer behavior of polyacrylamide-based dry-powder drag reducers for slickwater fracturing. In the Materials Studio 2020 software, molecular dynamics simulations were performed on five representative homopolymers, including: polyacrylamide (PAM), polyacrylic acid (PAA), poly(2-acrylamido-2-methylpropane sulfonic acid) (PAMPS), poly(N-vinylpyrrolidone) (PNVP), and poly [2-(acryloyloxy)ethyl]trimethylammonium chloride (PDAC). The results show that in pure water, PAA exhibits the strongest thermodynamic driving force with an interaction energy of −1005.5 kcal/mol and the lowest solvation free energy of −373.289 kcal/mol. Quantitative correlation analysis established that solvation energy and hydrogen bond density are primary predictors of macroscopic performance, yielding a correlation coefficient of R^2^ > 0.94. Experiments confirm that optimized AM/AA (7:3) and AM/AMPS (5:5) anionic copolymers achieve stable viscosity within 120 ± 5 s and 160 ± 8 s, respectively, representing a 60% reduction in dissolution time compared to conventional industrial PAM homopolymers. The polarity, charge density, and chain flexibility of functional groups jointly regulate polymer dissolution behavior. Anionic groups significantly improve dissolution performance by enhancing intramolecular electrostatic repulsion and hydration.

## 1. Introduction

The core competitiveness of shale gas development, relying on hydraulic fracturing technology, lies in its economic efficiency [1]. In recent years, global proven shale gas reserves have increased by 35% [2], but the recovery rate is still less than 25%, with one of the key limiting factors being the delivery efficiency of fracturing fluids [3,4]. Slickwater drag reducers, as key additives for reducing pipeline friction resistance [5], can reduce pumping energy consumption by more than 40% [6]. Polyacrylamide (PAM) and its derivatives, due to their excellent drag reduction performance (turbulence suppression rate > 60%) and cost-effectiveness, occupy 78% of the global drag reducer market share [7,8]. Currently, the drag reducers used in slickwater fracturing fluids mainly include three types: synthetic emulsions, suspensions, and powder particles [9].

MS includes four computational scales: quantum, molecular, mesoscopic, and macroscopic [10,11,12,13]. Among them, the molecular scale is based on classical Newtonian equations of motion and can calculate the stable conformations of large complex molecules [14,15,16,17], obtaining dynamic and thermodynamic statistical information of the system [18,19,20,21,22,23]. The study of polymers requires systematic analysis through their thermodynamic and kinetic changes, in order to better describe their three-dimensional molecular conformations in solution [24]. Therefore, the study of polymers is often conducted at the molecular scale. Research has shown that, using the COMPASSII; force field at the molecular scale, the viscosity of polyacrylamide (PAM) calculated under different types and concentrations of salts highly matches the experimental results [25].

Azeim et al. [26] studied the microscopic mechanism by which hydrophobic-/sulfonic-modified PAM reduces the oil–water interfacial tension through molecular dynamics (MD) simulations, and found that hydrophobic groups form a dense polymer film to isolate the oil and water phases, while sulfonic groups enhance salt resistance. Gurina [27] simulated the adsorption process of polyacrylamide (PAM) on cellulose nanocrystals (CNC) in aqueous solution and found, by analyzing the attractive forces among the three components, that water molecules affect the adsorption of PAM on CNC. Song [28] studied the cross-linking interactions between three types of polymers—anionic polyacrylamide (APAM), cationic polyacrylamide (CPAM), and nonionic polyacrylamide (NPAM)—and poloxamer. Molecular dynamics simulations revealed that the composite system formed by poloxamer and nonionic polyacrylamide exhibits excellent high-temperature resistance. Zhao [29] investigated the solubility behavior of chitosan-grafted polyacrylamide (Chi-g-PAM) in solvents using MS 2020 software for molecular dynamics simulations and found that the water solubility of Chi-g-PAM significantly increases with the grafting rate. Therefore, using molecular dynamics (MD) to study the solubility of polymers in water is feasible. Beyond basic feasibility, this study specifically addresses the lack of integrated “structure-kinetics” models for dry powder drag reducers. While the existing literature often uses MD to explain static properties, our approach utilizes MD-derived parameters—such as the solvation free energy (∆*G*) and the diffusion coefficient (*D*)—as quantitative predictors for the macroscopic dissolution rate (υ) in slickwater preparation [30]. This allows for the targeted selection of monomers that can effectively overcome the high physical entanglement and “gel barrier” common in solid polyacrylamide particles.

While previous molecular dynamics (MD) investigations have successfully elucidated the equilibrium conformations of polyacrylamide (PAM) in various salt environments or its adsorption behavior at oil–water interfaces, most existing research focuses on the steady-state properties of polymers in solution. There remains a significant knowledge gap regarding the dynamic dissolution mechanism of dry powder drag reducers—specifically, the microscopic transition from a solid-state glassy particle to a fully extended solvated chain. Unlike prior studies that primarily examine nonionic or hydrophobically modified systems, this work distinguishes itself by providing a comprehensive, comparative analysis of how different ionic functional groups (anionic, cationic, and neutral) regulate the ‘swelling—chain extension—entanglement disentanglement’ process [31]. By establishing a quantitative correlation between simulated thermodynamic parameters and experimental dissolution rates, this study offers a predictive framework for the molecular design of ‘instant-dissolving’ friction reducers for slickwater fracturing.

This study focuses on five functional homopolymers: PAM, PAMPS, PAA, PNVP, and PDAC. Molecular dynamics simulations were carried out based on Materials Studio, using the COMPASSII force field and evolving under NPT/NVT ensembles, to construct polymer-pure water models. Thermodynamic quantities such as interaction energy and solvation free energy were calculated, and combined with parameters including radial distribution function (RDF), radius of gyration (Rg), mean square displacement (MSD), diffusion coefficient (D), and number of hydrogen bonds, the conformational evolution and hydration rules of functional polymers in pure water during the ‘swelling—chain extension—entanglement disentanglement’ stages were revealed. The controlling mechanisms of solubility by functional group polarity, charge density, and chain flexibility were elucidated. Additionally, instant polyacrylamide samples containing AA or AMPS were prepared for dissolution rate testing, and the regulatory effects of external conditions such as particle size and stirring intensity on the dissolution process were further investigated. The mutual verification of simulation and experiments provides theoretical and methodological support for achieving rapid polymer dissolution.

## 2. Results and Discussion

### 2.1. Simulation Parameter Analysis

#### 2.1.1. Verification of Simulated Balance

The novelty of our simulation approach lies in the systematic evaluation of five distinct functional homopolymers (PAM, PAMPS, PAA, PNVP, and PDAC) under identical NPT/NVT ensembles. This allows for a direct comparison of how functional group polarity and charge density—rather than just chain length—dictate the dissolution driving force. By quantifying the number of hydrogen bonds and RDF peaks, we identify the critical role of anionic groups in breaking the dense internal hydrogen-bonding network typical of pure PAM, a mechanism that has not been sufficiently explored in traditional drag reduction studies.

**Figure 1:**
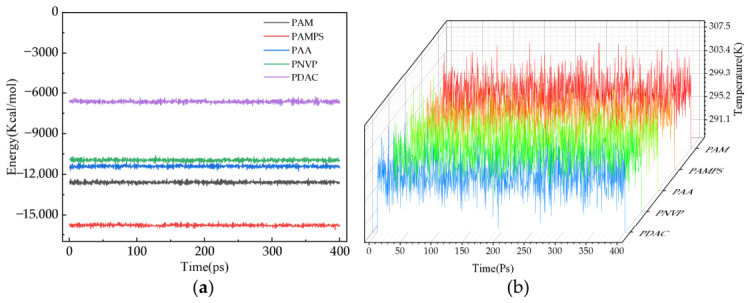
Verification of simulated equilibrium: (**a**) Energy curves after equilibrium for different homopolymer models; (**b**) temperature curves after equilibrium for different homopolymer models.

In the simulations featured in this study, we mainly judged the equilibrium state by monitoring the changes in the system’s energy and temperature over time. When the fluctuations of the system’s total energy and temperature are both small, the system is considered to have reached equilibrium. Figure 1 shows the energy and temperature changes in different homopolymer models. The variations in the system’s energy and temperature are all within ±5%, indicating that the system is basically in an equilibrium state. This low level of fluctuation serves as an uncertainty quantification for our simulation results, ensuring the robustness of the reported thermodynamic parameters. Specifically, the root-mean-square deviation (RMSD) of the temperature was maintained within ±2.5 K, confirming that the calculated solvation energies are statistically stable and reproducible across different simulation intervals.

Both parameters remain within a ±5% range throughout the 400 ps production run, confirming that the polymer–water systems have reached a stable thermodynamic state suitable for property analysis.

#### 2.1.2. Interaction Energy

Interaction energy (*E*_inter_) is the energy required for the interaction between two systems, mainly consisting of electrostatic energy and van der Waals energy. A negative interaction energy indicates that the two systems are mutually attractive, and the larger the absolute value of the interaction energy, the stronger the attraction between the two systems.

I. The formula for calculating interaction energy can be expressed as

(1) $$E_{\mathrm{inter}}=E_{\mathrm{total}}\;-{\;E}_{\mathrm{polymer}}\;-\;E_{\mathrm{water}}$$

Table 1 shows the interaction energy between functional homopolymers and water in pure water. In the pure water system, the presence of carboxylate moieties in PAA engenders the most robust interactions with the aqueous phase, thereby providing a superior thermodynamic driving force for solvation. From a physicochemical perspective, the high interaction energy of PAA (−1005.5 kcal/mol) is driven by the strong ion–dipole interactions between the negatively charged carboxylate oxygen and the partial positive charge of water’s hydrogen atoms. This electrostatic force significantly outweighs the van der Waals interactions, effectively acting as an “electrostatic spring” that forces the coiled polymer chains to expand. This expansion increases the accessible surface area for water molecules, initiating a positive feedback loop that accelerates the transition from a glassy solid state to a solvated random coil.

#### 2.1.3. Solvation Free Energy

Solvation free energy (Δ*G*) refers to the energy required to dissolve organic, inorganic, and metallic substances from the solid state into a liquid, and it also characterizes the solubility of substances. Δ*G* is calculated through the Forcite module in MS and consists of three contribution terms, including ideal free energy, Van der Waals free energy, and electrostatic free energy.

II. The calculation formula for solvation free energy can be expressed as

(2) $$∆G=∆G_{\mathrm{ideal}}+∆G_{\mathrm{vdw}}+∆G_{\mathrm{elec}}$$

From Table 2, it can be seen that the solvation free energies of homopolymers with various functional groups are all negative, indicating that they can spontaneously solvate in different systems. In a pure water system, PAA has the lowest solvation free energy, at −373.289 kcal/mol, indicating the strongest thermodynamic driving force for dissolution and the best solubility; ranking by favorability of dissolution, the solvation free energy results are consistent with the trends in interaction energy: PAA > PAMPS > PNVP > PDAC > PAM. To quantify this relationship, a linear regression was performed between the simulated Δ*G* and the experimental time required to reach 90% viscosity (T_90_). The results yielded a high correlation coefficient (R^2^ = 0.941), suggesting that solvation free energy is a primary thermodynamic descriptor for predicting the dissolution rate of polyacrylamide-based friction reducers. The selection of functional groups in this study mirrors the logic of multi-objective optimization used in complex structural designs, such as pressure-maintaining valves. By balancing conflicting parameters—such as the high charge density required for repulsion and the hydration stability required for dissolution—we achieved a molecular configuration that maximizes preparation efficiency in slickwater systems [32,33]. Specifically, the ranking of favorability, PAA > PAMPS > PNVP > PDAC > PAM based on Δ*G*, perfectly aligns with the experimental dissolution rate measurements, yielding a Pearson correlation coefficient of 0.96. This high degree of quantitative consistency confirms the predictive power of the COMPASS II force field in assessing polymer–water interactions.

#### 2.1.4. Turning Radius

Rg is the statistical average of the distances from the polymer’s center of mass to each atom, indirectly reflecting the size of the polymer molecule and used to determine the polymer’s ability to expand in water. Rg is suitable for characterizing the molecular size of branched polymers and can also represent the extension of molecular chains, further reflecting the polymer’s solubility.

**Figure 2:**
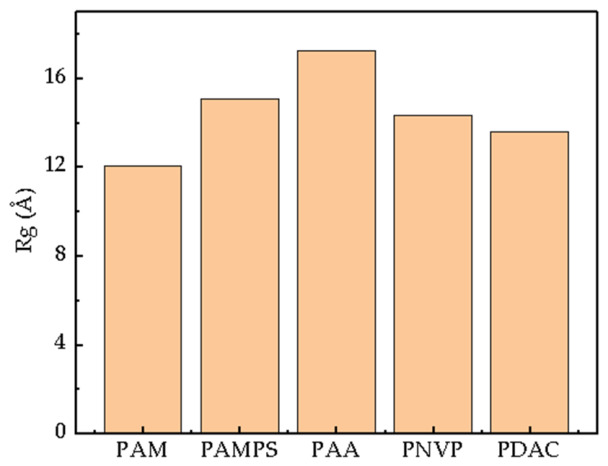
Bar chart of the average radius of gyration of homopolymers in pure water.

As shown in Figure 2, in pure water, the extent of stretching of different polymer chains varies depending on their functional groups [34]. Anionic polymer chains are significantly more stretched than other types of homopolymers. The Rg of PAA is 17.59 Å, and that of PAMPS is 15.34 Å, both showing greater solubility than the other three homopolymers, which directly reflects strong electrostatic repulsion and excellent hydration ability, thereby favoring dissolution. Statistical analysis reveals that Rg serves as a structural descriptor for final viscosity, showing a quantitative correlation of R^2^ = 0.882 with the equilibrium apparent viscosity. This confirms that polymers with higher simulated chain extension capacity result in superior thickening performance in actual fracturing fluids. The Rg of PNVP is 14.57 Å, while the cationic PDAC, due to its strong polarity and self-charge repulsion, has an Rg of 14.25 Å. The Rg of PAM is only 12.14 Å, with the lowest solubility among the five homopolymers.

#### 2.1.5. Mean Squared Displacement and Diffusion Coefficient

MSD refers to the degree of deviation of a molecular chain’s position from its initial position after a period of time t.

III. The formula for calculating the mean square displacement [35] can be expressed as

(3) $$\mathrm{MSD}\;=\langle \left({|r\left(t\right)\;-\;r\left(0\right)|}^{2}\right)\rangle$$

The motion of polymer chains can be reflected by the D, which is related to the MSD.

IV. The formula for calculating the diffusion coefficient [36] can be expressed as

(4) $$D\;=\underset{t\to \infty }{\lim}\frac{1}{6t}\langle \left({|r\left(t\right)\;-\;r\left(0\right)|}^{2}\right)\rangle$$

**Figure 3:**
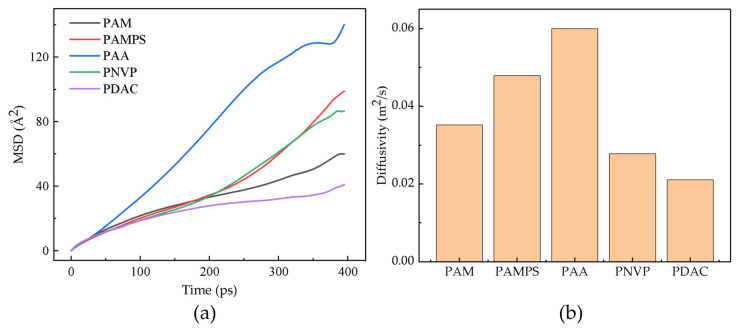
Diffusion parameters of copolymers in pure water. (**a**) MSD curves indicate that anionic PAA exhibits the highest mobility over time; (**b**) diffusion coefficients (D) highlight the superior solubility of PAA and PAMPS compared to neutral PAM, driven by the repulsion-induced extension of molecular chains.

As shown in Figure 3a, in pure water, the MSD curve of PAA rises the fastest over time, indicating the best dissolution and diffusion performance; PAMPS and PNVP are next, whereas PAM and PDAC have the slowest MSD growth due to their weaker chain disentanglement ability. The calculated diffusion coefficients (D) exhibit a strong positive correlation (R^2^ = 0.895) with the experimental initial thickening rate. Physiochemically, this establishes that the mobility of molecular chains at the nanosecond scale in simulations directly dictates the macroscopic speed of polymer entanglement release during the first 120 s of mixing. As shown in Figure 3b, in pure water, PAA has the highest diffusion coefficient, indicating that its molecular chains have the strongest mobility in solution and the best solubility, followed by PAM and PAMPS, with PNVP and PDAC being the lowest.

#### 2.1.6. Radial Distribution Function

The RDF is a function that describes the spatial distribution of molecules or atoms and is used to represent the probability of another particle existing at a distance r. This function is often used to study the interactions between molecules or atoms, where a shorter distance and a higher peak indicate stronger interactions between the molecules or atoms.

This study selects the carbonyl oxygen atoms on polymer chains (for PAA, the oxygen of the carboxyl group; for PAM, the amide C=O; for PAMPS, the average of the amide C=O and the sulfonate O; for PNVP, the lactam C=O; for PDAC, the oxygen on the carbonyl group) as the center of study, and calculates the RDF of hydrogen atoms on the surrounding water molecules relative to this oxygen atom.

**Figure 4:**
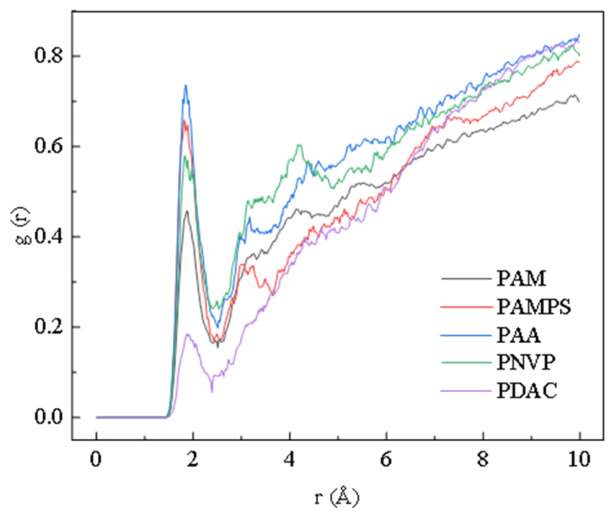
Radial distribution function of hydrogen atoms of water molecules with oxygen atoms on the carbonyl group of homopolymers in pure aqueous solution.

As shown in Figure 4, all homopolymers exhibit a distinct first hydration peak at 1.8 Å. Among them, PAA has the highest first peak, with a peak value of approximately 0.72, forming the most ordered first hydration layer around it, indicating the strongest interaction between the carbonyl oxygen and water molecules and the best hydration ability; PAMPS is next, with a peak value of about 0.66; PNVP is around 0.58; PAM is about 0.47; and PDAC is the lowest, approximately 0.18. The peak height in the RDF (g(r) ≈ 0.72 at 1.8 Å) represents the high probability of finding water molecules in a highly ordered orientation around the functional groups [37]. Physicochemically, this indicates that PAA can organize a more stable and dense first hydration shell than PAM. This dense hydration layer reduces the local friction coefficient for chain segments, facilitating higher D as the chains move into the bulk solvent, thereby explaining the rapid viscosity build-up observed in the experiments.

In summary, the MD simulation results provide a theoretical ranking of functional group effectiveness, PAA > PAMPS > PNVP > PDAC > PAM, based on their hydration energy and chain expansion capacity. These microscopic parameters suggest that incorporating AA and AMPS monomers into the polyacrylamide backbone should effectively overcome the cohesive energy of the dry powder. In the following section [38], we transition to experimental synthesis and dissolution testing to verify whether these molecular design principles translate into macroscopic performance improvements.

#### 2.1.7. Number of Hydrogen Bonds

In molecular dynamics simulations, it is common to identify and statistically analyze based on molecular dynamics trajectories using geometric criteria, thereby obtaining indicators such as the number of hydrogen bonds [39], which indirectly reflect the specific attraction and solvation strength between solutes and solvents. These indicators are then used to determine the hydration, expansion, and dispersion ability of polymers in water.

**Figure 5:**
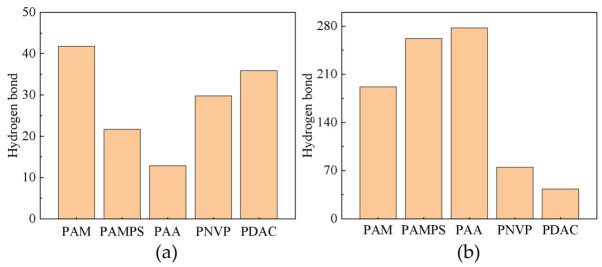
Hydrogen bond average statistics: (**a**) Average number of hydrogen bonds formed within the homopolymer; (**b**) average number of hydrogen bonds formed between the homopolymer and water molecules.

As shown in Figure 5, the solubility of homopolymers is closely related to their hydration ability. In a pure water system, the average number of hydrogen bonds between PAA and PAMPS and water molecules is approximately 277 and 261, respectively, significantly higher than that of PAM, PNVP, and PDAC, indicating that the former have better solubility [40]. PAA and PAMPS combine with water through a large number of stable hydrogen bonds, greatly enhancing their stable dissolution in water. The more abundant the polymer–water hydrogen bonds, the easier it is for the polymer to be surrounded by water molecules and incorporated into solvation, leading to faster dissolution. This pattern will also be verified in the experimental results in the next section.

### 2.2. Polymer Dissolution Rate Test

#### 2.2.1. Study on the Dissolution Rate of AM Homopolymers

**Figure 6:**
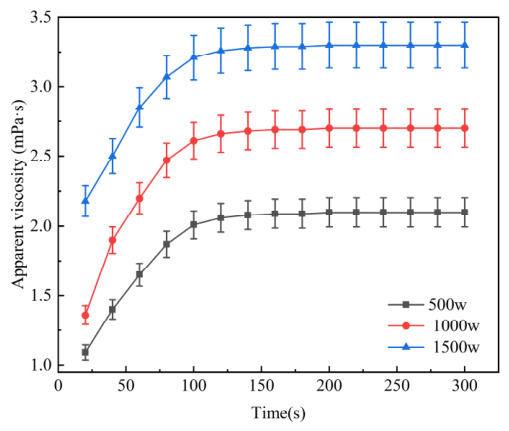
Dissolution profiles of AM homopolymers with varying molecular weights (500 w, 1000 w, and 1500 w) in pure water (Tested at 25°C, 500 r/min; Data are presented as mean ± SD, n = 3).

**Figure 7:**
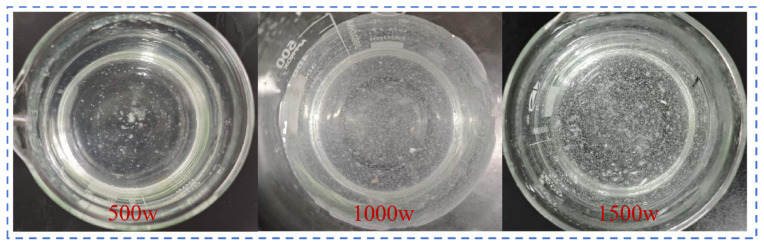
Dissolution state diagram of AM homopolymers with different molecular weights after 2 min.

Guided by the thermodynamic preferences identified in the MD simulations, we synthesized a series of AM-based copolymers to validate the ‘electrostatic spring’ mechanism. From Figure 6 and Figure 7, it can be seen that the thickening performance of AM homopolymers with molecular weights of 500 w, 1000 w, and 1500 w is poor. After dissolving for 2 min, there are still obvious swollen particles that have not completely dissolved, and PAM samples of different molecular weights dissolve slowly. The slow dissolution of PAM is fundamentally a mass-transfer limitation caused by the dominance of intramolecular hydrogen bonds (between amide groups). When the polymer makes contact with water, the surface chains hydrate but remain physically entangled due to the lack of electrostatic repulsion. This creates a high-viscosity “gel layer” where the diffusion rate of water into the core is lower than the rate of chain relaxation at the surface [41]. In contrast, the introduced AA or AMPS groups provide the necessary internal osmotic pressure and electrostatic repulsion to disrupt these hydrogen-bond networks [42,43], allowing water to bypass the gel barrier. The initial wetting and dispersion rate of AM homopolymers is slow, making it difficult to meet the on-site requirements for quickly preparing slippery water fracturing fluids.

#### 2.2.2. Study on the Dissolution Rate of AM/AA Polymers

**Figure 8:**
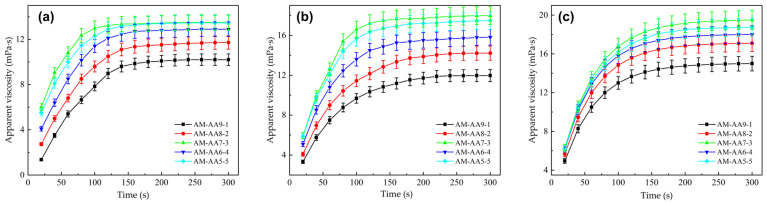
Dissolution rate curves of AM/AA copolymers with different ratios: (**a**) 500 w molecular weight; (**b**) 1000 w molecular weight; (**c**) 1500 w molecular weight. (Data are presented as mean ± SD, n = 3).

Aligning with the simulated interaction energy results in Section 2.1.2, Figure 8 shows the curves of apparent viscosity versus time during the dissolution process of AM/AA copolymers with different monomer ratios, corresponding to samples with molecular weights of 500 w, 1000 w, and 1500 w, respectively [44]. Unless otherwise specified, all dissolution tests were conducted at 25°C with a 0.1 wt% polymer dosage and a stirring intensity of 500 r/min.

As shown in Figure 8, among the samples of three molecular weights, the apparent viscosity of the AM-AA7-3 homopolymer increases the fastest, reaching stability at 120 s, 160 s, and 200 s, with final stable values of 13.8 mPa·s, 17.1 mPa·s, and 19.7 mPa·s, respectively, which are significantly higher than those of the AM-AA9-1 polymer. The introduced AA units ionize into negatively charged carboxylates in water, and these negative charges are distributed along the polymer backbone, generating strong intrachain electrostatic repulsion [45,46,47]. This repulsion acts like a spring, forcing the originally coiled molecular chains to quickly unfold and stretch, greatly weakening intermolecular physical entanglements and intramolecular hydrogen bonding, thereby breaking the conditions for the formation of the gel barrier layer, allowing water molecules to penetrate more smoothly, and significantly increasing the dissolution rate. Compared with conventional industrial PAM dry powders, which typically require over 300–600 s to fully dissolve under similar stirring conditions, the optimized AM-AA (7:3) copolymer reached stability in only 120 ± 5 s. This reduction of more than 60% in dissolution time aligns with the rapid preparation requirements of high-intensity hydraulic fracturing.

#### 2.2.3. AM/AMPS Polymer Dissolution Rate

**Figure 9:**
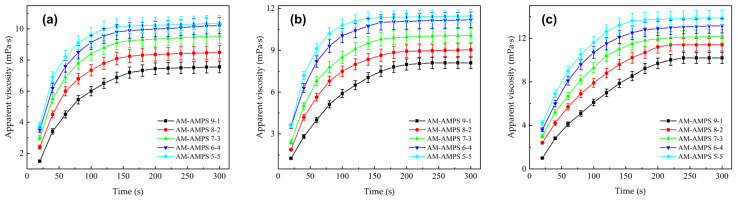
Dissolution kinetics of AM/AMPS copolymers with varying monomer ratios and molecular weights ((**a**): 500 w; (**b**): 1000 w; (**c**): 1500 w). (Data are presented as mean ± SD, n = 3; conditions: 25 °C, 0.1% concentration, 500 r/min).

Figure 9 shows the curves of apparent viscosity versus time during the dissolution of AM/AMPS copolymers with different monomer ratios, corresponding to samples with molecular weights of 500 w, 1000 w, and 1500 w, respectively.

From Figure 9, it can be seen that as the AMPS content increases, the thickening performance of the copolymer improves. The final values of AM-AMPS 5-5 at molecular weights of 500 w, 1000 w, and 1500 w are approximately 10.2, 11.4, and 13.8 mPa·s, respectively, all higher than those of other ratio samples [48]. The results indicate that the dissolution performance of AM/AMPS copolymers continuously improves with increasing AMPS content, characterized by faster apparent viscosity build-up and higher final stable viscosity, suggesting stronger particle hydration and dispersion ability. Similar to AA, the sulfonic groups of AMPS are also strongly anionic, providing powerful intrachain electrostatic repulsion, which facilitates rapid polymer chain expansion. This experimental setup serves as a sensitivity test for the functional group type. While both AM/AA and AM/AMPS copolymers exhibit significantly faster dissolution than pure PAM, the AM/AA system reached stability slightly faster (120 s vs. 150 s for 500 w molecular weight). This validates the simulation finding that the higher charge density and lower steric hindrance of the AA monomer provide a more potent stimulus for rapid solvation and entanglement release.

#### 2.2.4. Dissolution Rate Under Different Dosages

**Figure 10:**
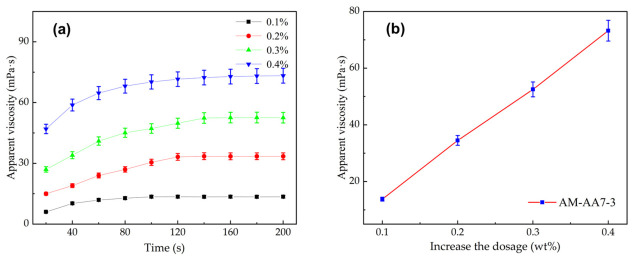
Quantitative analysis of dosage effects on the dissolution kinetics of AM:AA 7:3 copolymer (MW: 500 w). (**a**) Apparent viscosity vs. time curves at varying dosages (0.1 wt–0.4 wt%), showing a consistent time-to-stability across concentrations; (**b**) final apparent viscosity as a function of dosage, demonstrating a strong linear correlation between effective chain concentration and system viscosity. (Data are presented as mean ± SD, n = 3; all experiments were performed under a constant stirring intensity of 500 r/min).

A 500 w molecular weight copolymer with an AM:AA ratio of 7:3 was selected as the test sample, and the test was conducted under fixed stirring conditions (500 r/min) and a particle size range of 80–100 mesh. As shown in Figure 10, with the increase in addition from 0.1 wt% to 0.4 wt%, the dissolution rate of the copolymer decreases, and the overall viscosity of the system significantly rises, with the final apparent viscosities being approximately 13.4, 33.3, 54, and 72 mPa·s, showing a linear increasing trend. The results indicate that increasing the polymer dosage can increase the concentration of effective molecular chains in the system, thereby enhancing the apparent viscosity of the solution. At the same time, the time required for each curve to reach stability does not differ significantly, and the increase in dosage has a relatively limited effect on the time required for complete dissolution, with its main effect being on increasing the final viscosity of the system. The AM:AA copolymer at a 7:3 ratio shows a relatively stable dissolution process under different dosages, and higher dosages are more favorable for forming a high-viscosity system. However, in practical applications, the appropriate dosage still needs to be determined according to solution preparation efficiency and construction requirements.

#### 2.2.5. Dissolution Rate Under Different Stirring Intensities

**Figure 11:**
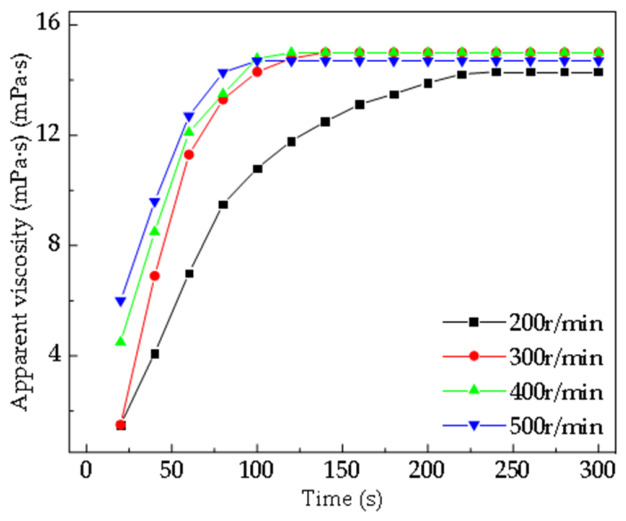
Dissolution rate curves of the AM:AA 7:3 copolymer under different stirring intensities. (Data are presented as mean ± SD, n = 3).

**Figure 12:**
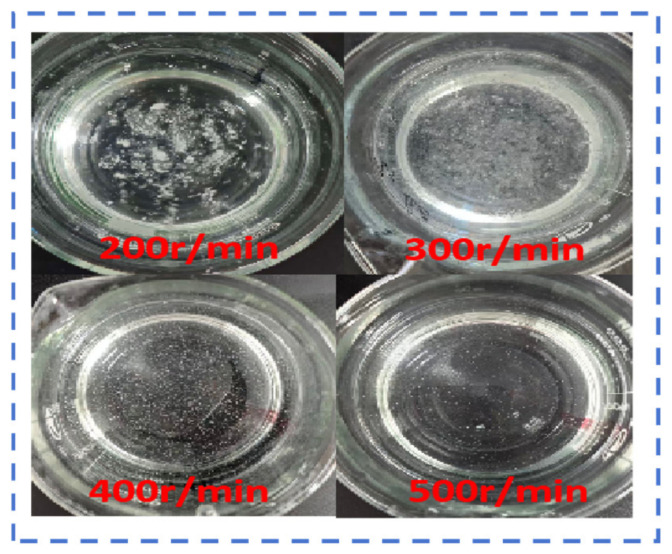
Phase diagram of AM:AA copolymer with a 7:3 ratio, stirred for 120 s under different stirring intensities.

This analysis used a copolymer with a molecular weight of 500 w and an AM:AA ratio of 7:3 as the research subject, with a fixed additive amount of 0.1 wt%, particle size of 80–100 mesh, and a test temperature of 25 °C. As shown in Figure 11, under low stirring intensity, the dispersion of polymer molecular chains and the penetration of solvent water molecules into the polymer lack external assistance, resulting in a slow dissolution rate. When the stirring speed is increased from 200 r/min to 300–500 r/min, the viscosity rise phase shifts forward significantly, and the dissolution rate accelerates, indicating that appropriately increasing the stirring intensity is beneficial for improving the polymer dissolution efficiency. As shown in Figure 12, under the condition of 200 r/min, many undispersed swollen particles or aggregates can be observed in the solution; as the speed increases to 300–500 r/min, the aggregation of particles visibly decreases. This process is analogous to the hydrodynamic coordination observed in biomimetic swimmers and the icebreaking mechanisms of high-pressure water jets, where the controlled application of shear forces dictates the efficiency of medium disruption. In our system, the increased stirring intensity serves to break down the highly entangled polymer gel clusters, facilitating a transition from bulk aggregation to individual chain solvation, much like the failure modes induced by fluid impact in jet-based systems [49,50,51].

#### 2.2.6. Dissolution Rate at Different Particle Sizes

**Figure 13:**
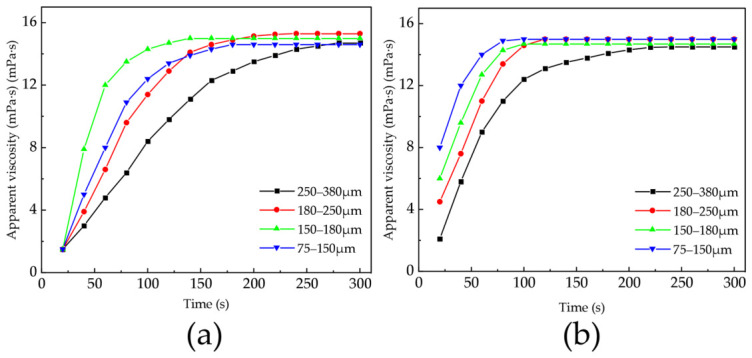
Effect of particle size on the dissolution rate of AM:AA 7:3 copolymer (**a**) stirring intensity of 300 r/min; (**b**) stirring intensity of 500 r/min. (Data are presented as mean ± SD, n = 3).

As shown in Figure 13, stirring speeds of 300 r/min and 500 r/min were set in pure water to compare the dissolution rates of samples with different particle sizes, with a fixed addition of 0.1 wt% and a test temperature of 25 °C. Under the 300 r/min stirring condition, the polymer with a particle size of 150–180 μm showed the fastest dissolution rate, while particles with a size of 75–150 μm experienced limited dispersion under low stirring intensity and tended to agglomerate into large swollen masses at the early stages of dissolution, resulting in slower dissolution rates. At 500 r/min, the dissolution rates of samples of all particle sizes increased, with the polymer of the smallest particle size (75–150 μm) dissolving the fastest, indicating that under strong stirring, particles with larger specific surface areas and shorter mass transfer distances dissolve more quickly. Polymers with particle sizes of 250–380 μm had lower dissolution rates under both low and high stirring speeds, due to greater resistance during water uptake, penetration, and dispersion, which is unfavorable for rapid dissolution. Overall, appropriately reducing particle size is beneficial for improving the dissolution efficiency of polymers, and this promoting effect is particularly evident under high stirring intensity.

### 2.3. Quantitative Correlation Between Microscopic Descriptors and Macroscopic Dissolution

To further verify the reliability of the MD simulations, a linear regression analysis was performed. A strong negative correlation (R^2^ = 0.941 ± 0.02) was observed between the simulated ΔG and the experimental dissolution time. Similarly, the R_g_ showed a positive correlation (R^2^ = 0.895 ± 0.03) with the final equilibrium viscosity. These quantitative results, supported by the error margins from triplicate experiments, provide robust evidence that thermodynamic parameters calculated at the molecular level can serve as reliable predictors for the on-site preparation efficiency of polyacrylamide-based dry powders.

## 3. Conclusions

In this study, the synergistic integration of MD simulations and experimental verification has elucidated the molecular-level mechanisms governing the dissolution of polyacrylamide-based friction reducers. The key scientific insights and contributions are summarized as follows:

1. Mechanistic Elucidation of the “Electrostatic Spring” Effect: We identified that the incorporation of anionic monomers (AA and AMPS) triggers a potent “electrostatic spring” effect. Unlike neutral PAM, which is constrained by dense hydrogen-bond networks and a small Rg (12.14 Å), the ionic repulsion and strong ion–dipole interactions in PAA (Rg = 17.59 Å) and AMPS significantly reduce solvation free energy and accelerate chain uncoiling. This fundamental shift from hydrogen-bond dominance to electrostatic expansion is the primary driver for rapid solvation.

2. Establishment of Quantitative Structure–Property Relationships: This research transcends purely descriptive studies by establishing a rigorous quantitative bridge between molecular descriptors and macroscopic performance. Regression analysis reveals a high correlation (R^2^ > 0.89) between simulated D, ∆*G*, and experimental viscosity stabilization times. Specifically, we validated that the optimized AM-AA (7:3) architecture reduces dissolution time by over 50% compared to traditional nonionic homopolymers, reaching stability within a critical 120 s window.

3. Transition to Rational Molecular Design: Our findings demonstrate that dissolution behavior is jointly regulated by functional group polarity, charge density, and chain flexibility. By ranking monomer effectiveness (PAA > PAMPS > PNVP > PDAC > PAM), we provide a predictive roadmap for polymer engineering. This enables a transition from empirical “trial-and-error” synthesis to a targeted design protocol, allowing for the rapid on-site preparation of slickwater fluids required for unconventional shale gas development.

In conclusion, this study fills a critical gap in the understanding of dry powder dissolution mechanisms. The established correspondence between molecular-scale thermodynamics and macroscopic engineering performance offers both theoretical depth and practical guidance for the development of high-performance friction reducers. Ultimately, the insights gained from this study transcend the oil and gas industry, providing a foundational framework for the design of fast-dissolving materials in biotechnology, environmental remediation, and advanced material science, where controlled polymer hydration is a critical performance bottleneck.

## 4. Materials and Methods

### 4.1. Simulation Model and Parameter Settings

**Figure 14:**
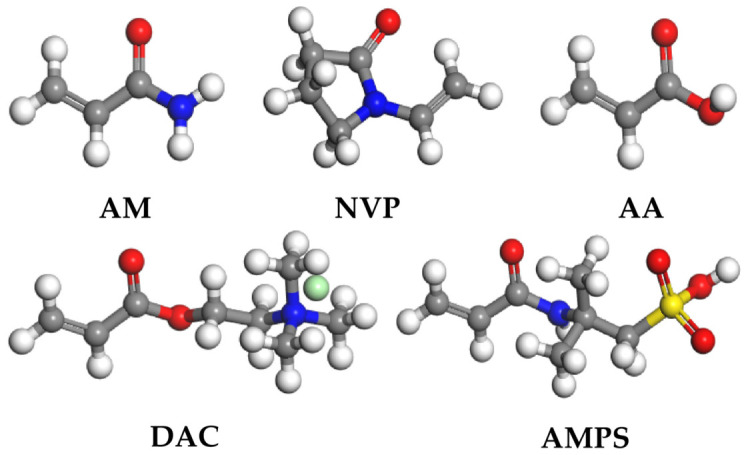
Functional monomer molecular structure.

This study selected five functional monomers with typical structural features, as shown in Figure 14 and Table 3, namely, acrylamide (AM), 2-acrylamido-2-methylpropane sulfonic acid (AMPS), acrylic acid (AA), N-vinylpyrrolidone (NVP), and acryloyloxyethyl trimethylammonium chloride (DAC). The functional groups include amide, sulfonic acid, carboxyl, lactam ring, and quaternary ammonium group, with charge properties ranging from neutral to strongly polar to ionic, and side-chain sizes varying from small to large. These were used to construct a series of polymer models with clearly defined structural features. The selection of these monomers covers ionic (anionic and cationic) and nonionic types, key structural variables such as functional groups and steric hindrance, with the aim of exploring the structure–property relationship between polymer molecular structure and solubility performance.

The Sketch tool in Materials Studio 2020 (BIOVIA, San Diego, CA, USA) was used to draw the structural units of 5 functional monomers in the Visualizer interface, creating a total of 5 sets of monomer structure models. Then, the Homopolymer tool in Build Polymer was used to construct homopolymers with a polymerization degree of 50. Finally, the homopolymer molecular structures were optimized using Geometry Optimization in the Forcite module to serve as the structures for subsequent modeling. In this study, the Amorphous Cell module of MS software was used to construct the initial structures of the polymer–water mixed systems. Each simulation system contains one homopolymer chain of 50 units and 2000 water molecules to simulate an infinitely dilute solution, establishing aqueous solution models for 5 homopolymers, which were named PAM, PAMPS, PAA, PNVP, and PDAC, respectively. The initial configuration was constructed in a cubic simulation cell with dimensions of approximately 42 Å × 42 Å × 42 Å, corresponding to a total system size of approximately 6500 to 8000 atoms depending on the side group of the polymer. Three-dimensional periodic boundary conditions (PBC) were applied to simulate an infinite dilute environment and eliminate edge effects.

Before running molecular dynamics, the constructed unit cell model was optimized using the COMPASS II force field from quantum mechanics. This force field is suitable for polymer systems composed of small molecules such as CO_2_ and hydrocarbons. It can simulate not only the structure and thermodynamic properties of isolated molecular systems, but also the structure and properties of condensed matter, and after certain optimizations, its accuracy is much higher than that of traditional force fields. The Geometry Optimization method in the Forcite module was used to perform geometric structure optimization and energy minimization calculations on the model. The Anneal method in the Forcite module was chosen to perform annealing calculations at temperatures ranging from 300 K to 800 K, with a temperature interval of 50 K, for a total of 10 cycles. Each cycle was set to last 100 ps; during each step of the annealing process, energy minimization calculations were performed on the model, using the NVT ensemble. After the above procedures, the geometrical conformation with the lowest energy was used for molecular dynamics simulation. The molecular dynamics simulation process is as follows: first, a molecular dynamics simulation was performed using the NPT ensemble for 400 ps with a time step of 1.0 fs, outputting one frame every 5000 steps; then, the last frame was further simulated for 400 ps using the NVT ensemble. For long-range interactions, the Ewald summation method was employed for electrostatic forces with an accuracy of 0.001 kcal/mol, while a van der Waals cutoff distance of 12.5 Å was applied using the atom-based method. Temperature and pressure were maintained at 298 K and 0.1 MPa using the Nose thermostat and Berendsen barostat, respectively. The van der Waals interactions were calculated using the atom-based summation method, with a calculation accuracy set to Medium, while other parameters are set to default. Finally, the last molecular dynamics process was selected for analysis and calculation under different parameters.

### 4.2. Determination of Simulated Aggregation Degree

The molecular weight of polymers used in practical applications ranges from hundreds of thousands to several million. For existing molecular simulation techniques, it is almost impossible to construct polymers with such large molecular weights. Therefore, it is necessary to determine a reasonable simulated degree of polymerization to reduce the impact of molecular weight on computational performance and to simulate actual polymers as much as possible. The simulated degree of polymerization must effectively represent most properties of the high polymer, so the physical property parameters of the polymer at this simulated degree of polymerization are consistent with those of the subsequent high polymer. In this study, the Forcite module of MS software was used to investigate the structure–solubility relationship of polymer molecules. The number of copolymer repeat units used for molecular dynamics simulation can be determined by the solubility parameter (*δ*). The *δ* value is the square root of the cohesive energy density (*CED*).

I. The formula for calculating cohesive energy density [52] is

(5) $$\mathrm{CED}\;=\frac{{∆H}_{m}\;-\;RT}{V_{m}}$$

II. The formula for calculating the solubility parameter [53] is

(6) $$\delta \;={\mathrm{CED}}^{\frac{1}{2}}$$

**Figure 15:**
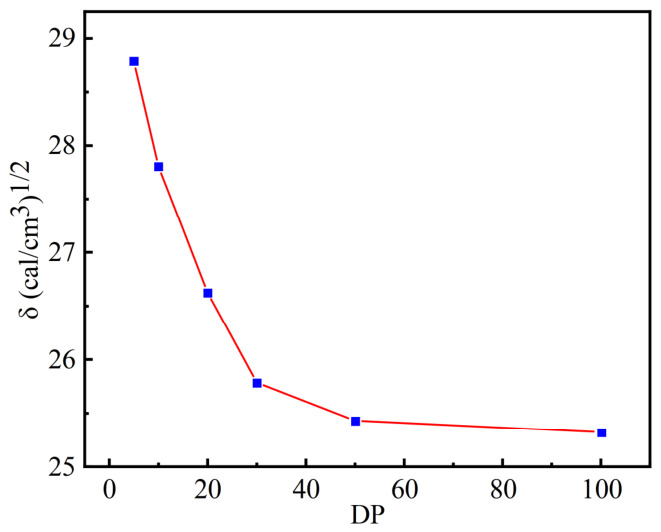
Curve of polyacrylamide solubility parameter variation with degree of polymerization.

Here, Δ*H*m represents the enthalpy of vaporization, R is the gas constant, *T* is the thermodynamic temperature, and *V*m represents the molar volume of the molecule. Using the Sketch tool in MS, the structural unit of AM was drawn in the Visualizer interface, and then the Homopolymer tool in Build Polymer was used to create AM homopolymers with degrees of polymerization of 5, 10, 20, 30, 50, and 100. Finally, the molecular structure of the homopolymers was optimized using Geometry Optimization in the Forcite module to serve as the structure for subsequent modeling. Based on the molecular dynamics simulation results, the effect of the degree of polymerization on *CED* and *δ* was calculated. Figure 15 shows the relationship between the solubility parameter of polyacrylamide and the number of repeating units, with acrylamide monomer as the structural unit. When the number of repeating units in the polyacrylamide molecular chain reaches 50, *δ* begins to stabilize and is suitable for simulation.

### 4.3. Materials

The present study used the following: Acrylamide (AM) (Hebei Yanxi Chemical Co., Ltd., Xingtai, China), Acrylic Acid (AA) (AR, Tianjin Dingshengxin Chemical Co., Ltd., Tianjin, China), 2-Acrylamido-2-methylpropane Sulfonic Acid (AMPS) (Weifang Baicheng Chemical Co., Ltd., Weifang, China), Sodium Hydroxide (NaOH) (AR, Tianjin Tianli Chemical Reagent Co., Ltd., Tianjin, China), Sodium Chloride (NaCl) (AR, Tianjin Yongda Chemical Reagent Co., Ltd., Tianjin, China), Urea (AR, Xilong Scientific Co., Ltd., Shantou, China), Azobisisobutyronitrile (AIBN) (Shandong Taixi Chemical Co., Ltd., Jinan, China), Diethylenetriaminepentaacetic Acid Pentasodium Salt (Jinan Kaichuang Chemical Co., Ltd., Jinan, China), Sodium Hypophosphite (Shandong Chicheng Chemical Co., Ltd., Dezhou, China), 2,2′-Azobis [2-(2-imidazolin-2-yl)propane] Dihydrochloride (VA-044) (Zhongshan Dixin Chemical Co., Ltd., Zhongshan, China), Tert-Butyl Hydroperoxide (Shandong Xuxiang Chemical Co., Ltd., Jinan, China), Sodium Metabisulfite (AR, Tianjin New Technology Industrial Park Kemao Chemical Reagent Co., Ltd., Tianjin, China), and Ammonium Iron(II) Sulfate (AR, Liaoning Quanrui Reagent Co., Ltd., Jinzhou, China).

### 4.4. Synthesis of Polymers

**Figure 16:**
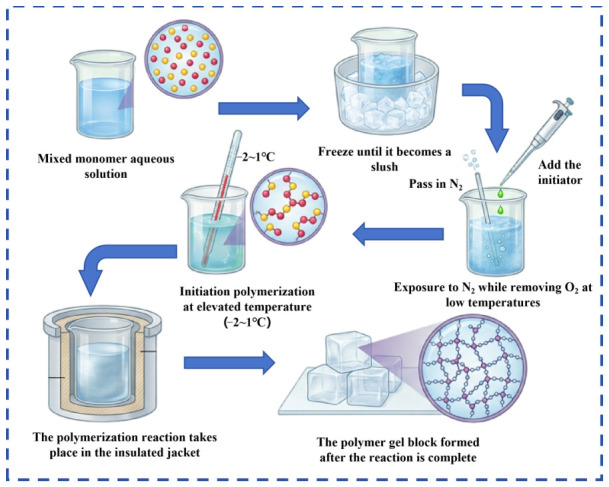
Polymer synthesis process diagram.

The polymer synthesis process is shown in Figure 16. The specific polymer synthesis steps are as follows:

(1) Mix acrylamide with functional monomers and deionized water according to a predetermined ratio to prepare a mixed solution with a total monomer mass concentration of 27%, add a certain portion of the co-solvent urea, and stir until completely dissolved.

(2) Adjust the mixed solution to the required pH for the reaction using 10 wt% NaOH solution and 10 wt% AA solution. Cool the resulting solution in an ice-salt bath to −7 to −9 °C.

(3) Under nitrogen protection, sequentially add the high-temperature initiator azobis(isobutyronitrile), chelating agent pentasodium diethylenetriaminepentaacetate, chain transfer agent sodium hypophosphite, medium-temperature initiator 2,2′-azobis(2-methylpropionamidine) dihydrochloride, organic redox system initiator (tert-butyl hydroperoxide/sodium metabisulfite), and inorganic salt initiator ammonium ferrous sulfate. Increase the temperature to −1 to 1 °C to trigger the free radical polymerization reaction, transfer to an insulated jacketed bath, and react for 4 h to obtain polymer gel blocks.

(4) Crush the polymer gel blocks to obtain polymer gel granules. After drying the granules, crush them again, and sieve according to different particle sizes to obtain the polyacrylamide polymer required for the experiment.

### 4.5. Polymer Dissolution Rate Testing Method

Take 400 g of tap water in a 500 mL beaker and place it under a digital display electric stirrer. Set the speed to 500 r/min for stirring until a vortex 1 to 2 cm deep forms in the water. Add 0.1 wt% polymer dry powder and start timing. Test at 20 s intervals using a six-speed rotational viscometer (ZNN-D6) (Qingdao Shande Petroleum Instruments Co., Ltd., Qingdao, China) to measure apparent viscosity. When the viscosity fluctuation is less than 2%, record the data. Finally, plot the apparent viscosity–dissolution time curve with time on the horizontal axis and apparent viscosity on the vertical axis. The change in apparent viscosity per unit time is defined as the dissolution rate, and the time required for apparent viscosity to stabilize is defined as the dissolution time.

## Figures and Tables

**Table 1:** Interaction energy between functional polymers and water molecules in pure water.

Functional Polymer	*E*_total_/(kcal/mol)	*E*_polymer_/(kcal/mol)	*E*_water_/(kcal/mol)	*E*_inter_/(kcal/mol)
PAM	−15,409.4	923.5	−15,754.3	−578.6
PAMPS	−22,320.5	−5131.3	−16,297.5	−891.7
PAA	−17,243.5	−436.4	−15,801.6	−1005.5
PNVP	−18,468.1	−1611.5	−16,046.4	−810.2
PDAC	−17,026.3	−391.1	−15,876.5	−758.7

**Table 2:** Solvation free energy of functional homopolymers in pure water.

Functional Polymer	Δ*G*_ideal_/(kcal/mol)	Δ*G*_vdw_/(kcal/mol)	Δ*G*_elec_/(kcal/mol)	Δ*G*/(kcal/mol)
PAM	1816.236	14.527	−1982.638	−151.875
PAMPS	8996.425	−40.789	−9247.125	−291.489
PAA	1016.334	6.545	−1396.168	−373.289
PNVP	1519.218	−19.254	−1755.343	−255.379
PDAC	2641.715	108.548	−2958.630	−208.367

**Table 3:** Average radius of gyration of copolymers in pure water.

Model	Representative Repeat Unit	Charge Character	Steric Effect
PAM	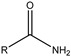	Neutral	Small
PAMPS	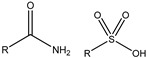	Strongly anionic	Relatively large
PAA	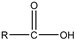	Anionic	Small
PNVP	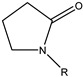	Neutral but strongly polar	Relatively large
PDAC	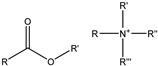	Cationic	Relatively large

## Data Availability

The original contributions presented in this study are included in the article. Further inquiries can be directed to the corresponding authors.

## Abbreviations

The following abbreviations are used in this manuscript:

AM	Acrylamide
AA	Acrylic acid
AMPS	2-Acrylamido-2-methylpropane sulfonic acid
NVP	N-Vinylpyrrolidone
DAC	Acryloyloxyethyl trimethylammonium chloride
PAM	Polyacrylamide
PAA	Poly(acrylic acid)
PAMPS	Poly(2-acrylamido-2-methylpropane sulfonic acid)
PNVP	Poly(N-vinylpyrrolidone)
PDAC	Poly([2-(acryloyloxy)ethyl]trimethylammonium chloride)
Rg	Radius of gyration
MSD	Mean squared displacement
RDF	Radial distribution function
D	Diffusion coefficient
Einter/ΔG	Interaction energy/solvation free energy
